# Engaging the community of women living with HIV to tailor and accelerate ARV research for pregnant and breastfeeding women

**DOI:** 10.1002/jia2.25920

**Published:** 2022-07-19

**Authors:** Polly Clayden, Jennifer M. Zech, Cadi Irvine, Imelda C. Mahaka, Angelina Namiba

**Affiliations:** ^1^ HIV i‐Base London UK; ^2^ ICAP at Columbia University New York City New York USA; ^3^ Department of Global HIV, Hepatitis and Sexually Transmitted Infections Programmes World Health Organization Gijón Spain; ^4^ Pangaea Zimbabwe AIDS Trust (PZAT) Harare Zimbabwe; ^5^ 4M Mentor Mothers Network London UK

**Keywords:** pregnancy, public health, community engagement, HIV, research, choice

1

Since the beginning of the HIV epidemic, community engagement and activism have played a critical role in shaping clinical research and drug development. Community engagement in research is a complex and interactive relationship between researchers, policymakers and the community. Such engagement has been acknowledged as a critical component of successful outcomes by key health agencies, including the World Health Organization (WHO) [1].

Good practice involves participants and advocates as partners rather than merely trial subjects or users of the intervention [2]. When done effectively, engagement should lead to the community becoming increasingly aware of and involved in research activities, processes and decision making. Given the challenges and barriers to studying antiretroviral (ARV) drugs in women of childbearing potential and pregnant women—who are not only thinking about their own health, but also the health of their child, and balancing the risks and benefits even in the face of uncertainty—engaging the community of women living with HIV is critical to accelerating ARV research in this population [3].

WHO and the International Maternal, Pediatric, Adolescent AIDS Clinical Trials (IMPAACT) Network convened a workshop on “Approaches to Enhance and Accelerate Study of New Drugs for HIV and Associated Infections in Pregnant Women,” which included seven women—who were invited due to their roles in advocacy for pregnant women with HIV—to participate in two community panels [4]. This Viewpoint is based on discussions that took place during and around these panels [5]. The workshop focused on pre‐licensure trials and studies to determine dosing and safety in pregnant women. Most of our discussions and recommendations are appropriate for all research, while some are particular for trials of new agents.

Through these discussions, we identified five key recommendations: (1) pregnant women and women who become pregnant have the right to make their own choice about participating in research, (2) clear and understandable information must be shared for informed decision making, (3) pregnant women should be included in research in a timely manner, (4) contraception should not be a prerequisite to study participation and women who become pregnant should be able to stay on study drug and (5) pregnant women should be engaged across the lifecycle of trials (Figure 1).

**Figure 1:**
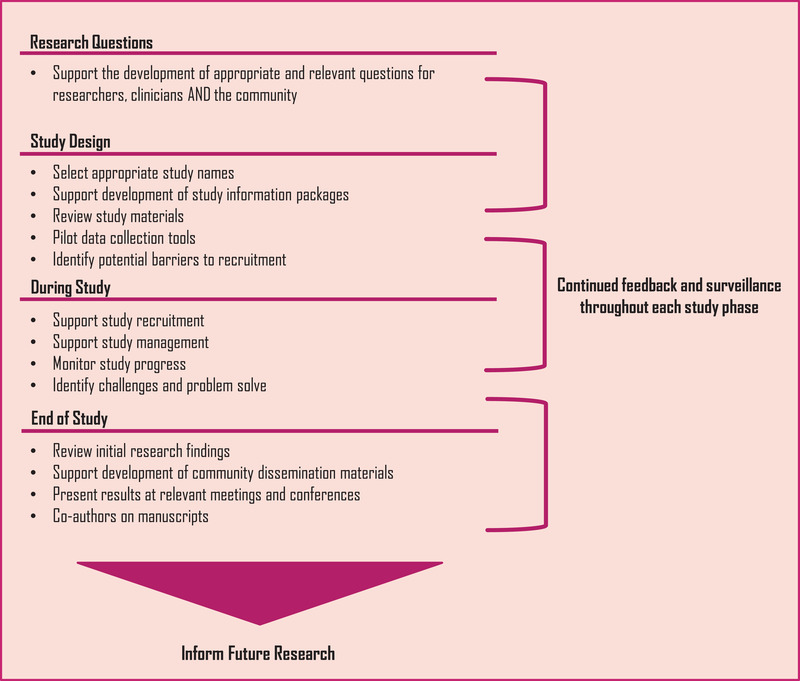
When and how to engage pregnant women in research studies.

All participants, and those considering enrolment, are capable of understanding the risks and benefits of a trial when given the right information. There are risks and benefits to all research and pregnant women and those who become pregnant during the trial should be given the opportunity to weigh the potential risks and benefits and decide for themselves if participation is right for them and their child. Women have the right to make their own informed decisions about participating in research.

**Key informant voices**

- We are not asking for pregnant women to be exposed to undue risks, but when we have opportunities to obtain information on a drug during pregnancy, such as when women become pregnant during trials or after safety has been ascertained, pregnant women should not be denied inclusion, especially for drugs that will be beneficial to them.
- Women are completely capable of making their own decisions to use contraception, given the correct information. They should be given the opportunity to make choices about their bodies and also about their children and wanting to participate in something. Obviously, as a woman, I wouldn't be willing to risk my child's safety, but if the drug is going to be used in other people when they are pregnant, then why not allow us to decide as soon as the medicine is proven to be safe and effective for use? Women who are willing and well‐informed should be allowed to participate in clinical trials without contraception.
- Clinical trials and the work of researchers can also benefit from including women not only from the beginning of the trial but also throughout the lifecycle of trials.

Research information must be shared by the research group with the participants in a clear and understandable manner, outlining their rights and be transparent about the risks and benefits. It should also be clear why and how participation in research will benefit other women around the world in the future. The information needs to be presented appropriately for different literacy levels, in local languages and be sensitive to gender identity where relevant. Peer support and counselling must be offered as part of the study information package as well as during participation and beyond. Study teams should consider using diverse formats (e.g. infographics, animation and live videos) to present information to the community in order to reach all potential participants and influencers. It is critical to partner with communities and peer educators to develop and pilot the content and dissemination methods of study information packages.

Problems have occurred when communities have not received clear information or been sufficiently engaged. A recent example being the safety signal with dolutegravir, identified in the Tsepamo study, initially suggesting an elevated risk of neural tube defects among babies exposed to this drug [6]. The response to the initial available data was made out of an abundance of caution, supported at the time by multiple experts and agencies. However, this risk was sometimes misinterpreted, misunderstood and poorly but widely communicated to women and the community at large. Although new guidance was subsequently developed when the safety signal attenuated, in some facilities, health workers refused to provide women with dolutegravir, even after the risk was demonstrated to be negligible. After community‐informed national campaigns, treatment literacy programmes and advocacy campaigns, women were empowered to demand this drug and make informed choices [7]. Earlier involvement of the community of women living with HIV could have helped better understanding and engagement with the complexity of the issue.

Research, including pre‐licensure studies, needs to move away from the presumptive exclusion of pregnant women in clinical trials with promising early data. Without inclusion in clinical trials, pregnant women will receive new drugs in a way that is uncontrolled and unconsented, placing them at risk for incorrect dosing or unknown adverse effects.

Pregnant women should not be exposed to undue risks, but when there are opportunities to obtain information on a drug during pregnancy, such as when women become pregnant during trials or after safety has been ascertained, pregnant women should not be denied inclusion, especially for drugs that will be beneficial to them. Pregnant women are properly monitored in trials, the alternative is to receive the drug after approval with no relevant data, no monitoring or follow up.

Where a drug could be highly beneficial for pregnant women, delaying rollout to this population means postponing newer and improved treatments—such as those with fewer side effects or longer‐acting formulations. Participating in a clinical trial also has many benefits, including receiving extensive support, counselling and medical attention, which a participant would not have had access to outside the study setting. Within a study, it can be easier to identify drug‐associated adverse events and to switch to a more suitable drug in a timely fashion.

The contraception requirement for participation in many trials is frequently viewed as unfair by the community. Women are capable of making their own decisions to use contraception, given the correct information. Women who are willing and well‐informed should be allowed to participate in clinical trials without contraception.

Similarly, women who become pregnant on trials should be given the option to make an informed choice to stay on study drug and contribute to pregnancy research and safety data. Each participant's case is different and broad exclusion criteria restrict the possibility for women to continue participation in a study that could be beneficial to both themselves and their child. To support women to make these decisions, they must be well‐counselled and informed.

Clinical trials and the work of researchers can also benefit from including women not only from the beginning of the trial but also throughout the lifecycle of trials. Figure 1 offers practical ways that community engagement can happen and start as early as possible in research.

Overall, we conclude that women (including pregnant women) are not a “niche,” “‘key” or “special” population. They are the population, and research should recognize this and adapt accordingly. Women should be protected *through* research not *by* research [8].

Pregnant women want to make their own informed decision about participating/continuing to participate in clinical trials. With the appropriate information and support, women should have autonomy to decide. Meaningful engagement of women of childbearing potential affected by HIV in ALL stages of clinical trials is critical; from the identification of research questions through the study design, recruitment, conduct and dissemination of results. This involvement is not only beneficial to community members of women living with HIV, but to the research community as whole and should be viewed as an asset.

## COMPETING INTERESTS

The authors have no competing interests.

## AUTHORS’ CONTRIBUTIONS

PC wrote the first draft. JZ wrote the first outline, based on panel discussions. PC, JZ and CI contributed to the writing of the final manuscript. All authors reviewed, commented and edited this Viewpoint.
